# Acute hepatic porphyria in Denmark; a retrospective study

**DOI:** 10.1186/s13023-025-03536-3

**Published:** 2025-02-28

**Authors:** Magnus Emil Ulrich Wagner, Morten Frost, Jan Frystyk

**Affiliations:** 1 Department of Endocrinology, Odense University Hospital, Kløvervænget 6, Entrance 93, Level 4, DK-5000 Odense C, Denmark; 2 Department of Clinical Research, Faculty of Health Sciences, University of Southern Denmark, Odense, Denmark

**Keywords:** AIP, VP and HCP, PBG

## Abstract

**Background:**

Acute hepatic porphyria (AHP) constitutes a class of rare diseases caused by reduced function in enzymes of the heme-biosynthetic pathway. AHP includes acute intermittent porphyria (AIP), hereditary coproporphyria (HCP), variegate porphyria (VP) and the extremely rare δ-aminolevulinic-dehydrase deficiency porphyria (ADP). This retrospective study describes characteristics of the Danish AHP patient population.

**Methods:**

Department of Endocrinology at Odense University Hospital serves as national AHP center. We performed a 5-year retrospective description of our AHP cohort using electronic patient journals. We included general symptoms, number of acute attacks, hospitalization rates, long-term sequelae and symptoms, and grouped patients according to creatinine-adjusted urinary baseline excretion (i.e., outside attacks) of the porphyrin precursor porphobilinogen (PBG) in normal-, moderate- and high-excretion and unknown.

**Results:**

The cohort contained 129 AHP patients, hereof 100 AIP, 12 HCP and 17 VP. Median age was 46.3 (32.1–62.0) years, and 85 (65.9%) were female. During the 5-years, 38 (29.5%) patients experienced symptoms. Hereof, 20 patients were hospitalized with acute attacks or chronic symptoms and treated with human hemin (*n* = 14). Most frequently reported symptoms were abdominal pain, nausea, vomiting, and neurological disturbances. Symptoms were more common in patients with high PBG baseline excretion (*n* = 39) as compared to those with moderate (*n* = 31) or normal (*n* = 40) PBG excretion (*p* = 0.002). Furthermore, females dominated the symptomatic group (68.4%).

**Conclusion:**

As reported internationally, AHP is more commonly diagnosed and symptomatic in women, and AIP was the most frequent AHP subtype. Those with an elevated urinary baseline PBG secretion were more likely to report AHP-related symptoms.

## Introduction

Acute hepatic porphyria (AHP) constitutes a group of rare metabolic diseases caused by a genetic mutation in one of four genes encoding enzymes of the heme biosynthesis, resulting in partial enzyme deficiency [1–3]. Due to enzyme deficiency, neurotoxic intermediates accumulate upstream of the defective enzyme when heme synthesis is increased [1, 4] There are four subtypes of AHP. Acute intermittent porphyria (AIP), variegate porphyria (VP) and hereditary coproporphyria (HCP), are autosomal dominant (AD) inherited whereas δ-aminolevulinic-dehydrase deficiency porphyria (ADP) is inherited in an autosomal recessive manner. The latter is extremely rare [5], and will not be further discussed. The penetrance of AHP is low, and most carriers with a pathogenic variant in the AHP genes never experience acute attacks but remain free of symptoms. The penetrance of symptomatic disease is about 1% in carriers of AIP mutations, increasing to > 20% in families with symptomatic AIP patients [6]. The penetrance of pathogenic HCP and VP variants is reported to be lower than for AIP [1, 4].

**Fig. 1:**
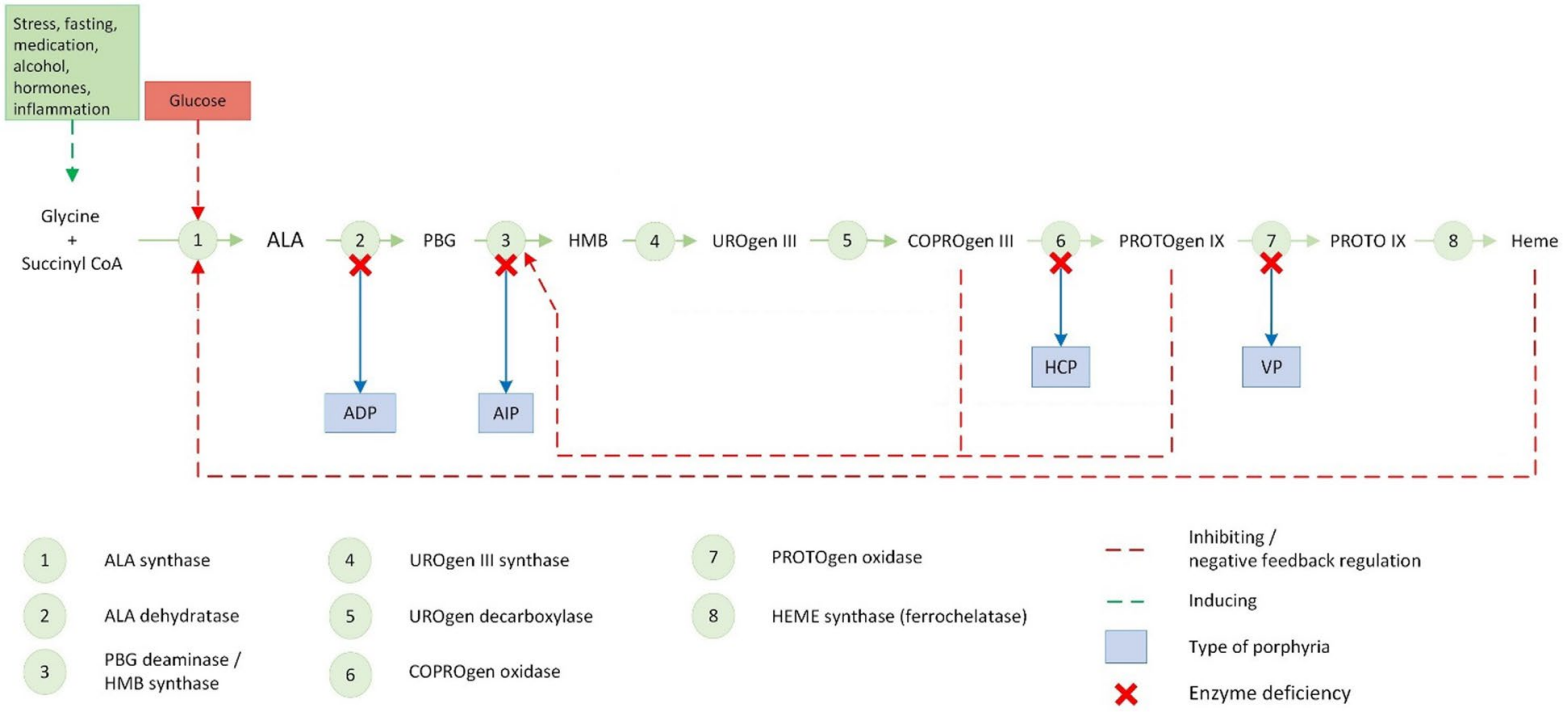
The pathway of the heme biosynthesis. ALA (δ-aminolevulinic acid), PBG (porphobilinogen), HMB (hydroxymethylbilane), URO (uroporphyrin), COPRO (coproporphyrin), PROTO (protoporphyrin), ADP (δ-aminolevulinic-dehydrase deficiency porphyria), AIP (acute intermittent porphyria), HCP (hereditary coproporphyria), VP (variegate porphyria) [2, 3, 7]. The precipitating factors are shown in the green box [19]

Heme intermediates as shown in Fig. 1 include porphyrin precursors δ-aminolevulinic acid (ALA) and porphobilinogen (PBG) which are considered neurotoxic. Accumulation of ALA and PBG is the main cause of episodic neurovisceral attacks which most commonly include severe generalized abdominal pain and gastrointestinal symptoms such as nausea, vomiting, and constipation. Moreover, patients with VP and HCP may experience AHP-related skin lesions due to accumulation of light-absorbing porphyrins in the skin [5, 7]. An attack develops over hours to days [1, 8]. Neurological symptoms are present in up to 70% of patients experiencing an acute attack and involves the peripheral, central, and autonomous nerve system [7, 9, 10]. Due to the non-specific symptoms and findings of an acute attack, there are often considerable delay in the diagnosis of patients suffering from AHP [5, 7, 11]. The diagnosis of symptomatic AHP involves biochemical testing, where an elevated urinary concentration of PBG is the hallmark of an acute attack, during which the urinary concentration of PBG is usually 5–10 times higher than the upper normal limit [7, 10, 12]. Some of the symptomatic AIP carriers, varying from 15 to 44% in previous studies [7, 10], will have a normal urine analysis when asymptomatic.

The excretion of the precursors PBG and ALA and the intermediate uroporphyrin are exclusively by renal filtration, whereas coproporphyrin is excreted both in feces and urine, and protoporphyrin in feces only [1]. Due to low penetrance of the disease [7], the gold standard of diagnosing symptomatic AHP relies on identification of an elevated urinary concentration of PBG, which is usually accompanied by elevated ALA concentrations. Both precursors can be measured with high sensitivity and specificity by quantitative column assays or mass spectrometry [7]. Subsequently, AHP subclassification can be supported by genetic mutation analysis, which identifies disease-promoting mutations in 95–99% of the cases [7, 13]. Importantly, heme exerts negative feedback on the first and rate-limiting step of the heme synthesis. Accordingly, administration of exogenous hemin is used as management of acute attacks.

Patients with latent or symptomatic disease have a greater risk of hypertension, chronic renal failure, and primary hepatocellular carcinoma (HCC) [7, 14, 15]. The risk of HCC is probably highest in patients with a history of active disease (recurrent attacks and/or chronically elevated ALA- and PBG-levels). According to current recommendations, patients ≥ 50 years of age are offered regular liver ultrasonography to screen for HCC [6, 7, 15].

The Department of Endocrinology at Odense University Hospital serves as the national center for patients with AHP in Denmark. The Danish population of AHP patients was originally described in 1980 [16], however, biochemical tests and DNA analyses have improved since then. Therefore, the aim of this study was to characterize AHP patients referred to the Danish national center including their level of excretion of porphyria precursors and risk of having symptoms.

## Methods

### Study population

We identified symptomatic individuals and asymptomatic carriers attending follow-up at Department of Endocrinology at Odense University Hospital. Patients with symptomatic AHP were identified by the presence of elevated urinary PBG combined with either acute attacks or chronic symptoms, whereas asymptomatic carriers were identified by the presence of a predisposing mutation in the HMBS, CPOX or PPOX gene.

We grouped patients according to their highest documented urinary concentration of PBG (U-PBG/creatinine) outside attacks (i.e., baseline) during the last 5 years. In accordance with a previous study [15], patients were categorized in three groups: U-PBG-normal (patients with a U-PBG/creatinine level never exceeding the upper limit of normal (ULN); 0.8 mmol/mol), U-PBG-moderate (0.9–3.2 mmol/mol [i.e., maximum 4xULN]) and U-PBG-high (≥ 3.3 mmol/mol). Patients with no documented U-PBG concentration formed a fourth group: U-PBG-unknown. Urine concentrations of PBG and ALA were measured using a commercial kit as described by the manufacturer (Clin Easy Photometric Compete kits, product no 17200, from Recipe Chemical + Instruments, Munich, Germany).

### Data collection and management

Patient data were obtained from electronic patient journals after legal approval. The electronic patient journal system provides access to all communication with patients, reports on imaging, histological investigations, genetic investigations, and biochemistry as well as use of prescription medicine at time of the last visit to the center. The legislation limited us to include data for the last 5 years and to include data from our hospital’s patient journal system only. Hence, we did not have access to the Danish National Electronic Journal System. Consequently, some patients may have received hospital treatment for AHP without our knowledge. Data were stored at a logged and safe web-based collaborative platform, Share-Point (Microsoft Corporation 2022, Santa Rosa, CA, USA).

### Statistical analysis

Categorical variables are presented as numbers and percentages, and continuous variables as means and interquartile ranges (IQR). Because of the uneven distribution of patients in three AHP subgroups, (100 AIP, 12 HCP and 17 VP), HCP and VP patients were combined as one group to improve statistical power. We compared findings in AHP subtypes AIP vs. HCP + VP using Pearson’s chi-squared test. Based on differences in the three groups of known U-PBG excretion types, a subgroup analysis was performed. Data were not normal-distributed, and accordingly, we used non-parametric ANOVA, i.e., Kruskall-Wallis one-way analysis of variance. Pearson’s chi-squared test was used to test for differences between the three groups. The prevalence of AIP overall and symptomatic AIP were calculated from the number of citizens registered in Denmark primo 2022 (5.87 million) [17].

Microsoft Excel v. 16.60 (Microsoft Corporation 2022, Santa Rosa, CA, USA) was used to do the descriptive statistics. STATA Statistical Software Release 17 (StataCorp 2021, StataCorp LCC, College Station, TX, USA) was used to perform all the statistical analyses. P-values < 0.05 were considered statistically significant.

### Ethical aspects

Prior to start, the study was formally approved by the Legal Department at Odense University Hospital, and because of the retrospectivity of maximum five years, no further permissions were required to access the hospital’s electronic journal of the included patients. Furthermore, there was an approved data-management agreement. There were no conflicts of interest.

## Results

### The cohort

During the period from February 2017 to February 2022, 129 patients with newly diagnosed or established AHP attended our outpatient clinic (telephone or video contact, or face to face consultation) at Department of Endocrinology at Odense University Hospital. Characteristics of the AHP population are shown in Table 1. The mean age was 46.3 years and 85 of the patients (65.9%) were females. Of our AHP population, 100 patients (77.5%) had AIP, 12 patients (9.3%) HCP and 17 patients (13.2%) VP. Patients with HCP and patients with VP are considered as one group.

**Table 1:** Descriptive characteristics of the study population. Patients with HCP and VP are considered as one group due to low number of subjects

	All AHP’s	AIP	HCP + VP
Total, *n* [%]	129 (100.0%)	100 (77.5%)	29 (22.5%)
Female sex, *n* [%]	85 (65.9%)	65 (65.0%)	20 (69.0%)
Age, mean [IQR]	46.3 (32.1–62.0)	48.2 (33.5–63.0)	39.9 (21.9-54.1)
Excretion type [PBG]			
U-PBG normal, *n* [%]	40 (31.0%)	24 (24.0%)	16 (55.2%)
[≤ 0.8 mmol/mol]			
U-PBG moderate, *n* [%]	31 (24.0%)	25 (25.0%)	6 (20.7%)
[0.9–3.2 mmol/mol]			
U-PBG high, *n* [%]	39 (30.2%)	36 (36.0%)	3 (10.3%)
[≥ 3.3 mmol/mol]			
Unknown	19 (14.7%)	15 (15.0%)	4 (13.8%)
Excretion type [ALA]			
U-ALA normal, *n* [%]	83 (64.3%)	62 (62.0%)	21 (72.4%)
[≤ 5.0 mmol/mol]			
U-ALA moderate, *n* [%]	20 (15.5%)	16 (16.0%)	4 (13.8%)
[5.1–20.0 mmol/mol]			
U-ALA high, *n* [%]	7 (5.4%)	7 (7.0%)	–
[≥ 20.1 mmol/mol]			
U-ALA unknown, *n* [%]	19 (14.7%)	15 (15.0%)	4 (13.8%)
Asymptomatic, *n* [%]	91 (70.5%)	67 (67.0%)	24 (82.8%)
Symptomatic, *n* [%]	38 (29.5%)	33 (33.0%)	5 (17.2%)
Female sex, *n* [%]	26 (68.4%)	22 (66.7%)	4 (80.0%)
Hospitalization, *n* [%]	20 (15.5%)	18 (18.0%)	2 (6.9%)

During the 5-year period, 38 patients (29.5%) reported having symptoms attributed to AHP at least once, and hereof 26 (68.4%) were female. Thus, 30.6% (26 of 85) of all female AHP patients had symptoms, while 12 of 44 (27.7%) male AHP patients had symptoms. Based on our cohort, the overall prevalence for AHP in Denmark was calculated to 2.2:100,000, whereas the prevalence for symptomatic AIP was 0.6:100,000.

### Hospitalizations

Twenty patients (15.5%) were hospitalized one or more times due to AHP-related symptoms in the same period: 14 patients (70.0%) were hospitalized 1–4 times, whereas 6 patients were hospitalized ≥ 5 times (i.e. at least one annual admission). Eleven patients had one or more acute attacks requiring hospitalization and treatment with human hemin (Normosang®; Recordati Rare Diseases, France) for a minimum of 4 days, whereas 3 hospitalizations were due to prophylactic hemin treatment of chronic symptoms. In addition to hemin treatment, all patients also received intravenous glucose, 300–400 g per 24 h, as glucose inhibits the production of porphyrin precursors [18].

### Baseline levels of the urinary porphyria precursors PBG and ALA

Thirty-one patients (24.0%) had moderately elevated U-PBG/creatinine levels (0.9–3.2 mmol/mol) and 39 patients (30.2%) had high U-PBG/creatinine levels (≥ 3.3 mmol/mol). In 40 patients (31.0%), the U-PBG/creatinine level was below the ULN (U-PBG-normal). Data were missing (U-PBG-unknown) in 19 patients (14.7%). The characteristics of the U-PBG subgroup are shown in Table 2.

**Table 2:** Characteristics of the AHP population by urinary porphobilinogen [U-PBG]

	All AHP’s	U-PBG-normal	U-PBG-moderate	U-PBG-high	U-PBG-unknown
		[≤ 0.8 mmol/mol creatinine]	[0.9–3.2 mmol/mol creatinine]	[≥ 3.3 mmol /mol creatinine]	
Total, *n* [%]	129 (100.0%)	40 (31.0%)	31 (24.0%)	39 (30.2%)	19 (14.7%)
Female sex, *n* [%]	85 (65.9%)	20 (50.0%)	23 (74.2%)	31 (79.5%)	11 (57.9%)
Age, mean [IQR]	46.3 (32.1–62.0)	38.8 (21.1–56.8)	50.2 (43.1–62.6)	55.1 (41.5–68.4)	37.7 (22.5–52.2)
PBG concentration, mean [IQR] [mmol/mol creatinine]	6.3 (0.6–5.6)	0.5 (0.4–0.67	1.6 (1.1–2.0)	15.9 (4.9–22.5)	–
Asymptomatic, *n* [%]	91 (70.5%)	33 (82.5%)	23 (74.2%)	18 (46.2%)	17 (89.5%)
Symptomatic, *n* [%]	38 (29.5%)	7 (17.5%)	8 (25.8%)	21 (53.8%) *	2 (10.5%)
Hospitalization, *n* [%]	20 (15.5%)	4 (10.0%)	2 (6.5%)	13 (33.3%)	1 (5.3%)
Hemin treatment, *n* [%]	14 (10.9%)	1 (2.5%)	2 (6.5%)	10 (25.6%)	1 (5.3%)

^*^ = significantly different from U-PBG-normal (*p* = 0.001) and U-PBG-moderate (*p* = 0.018)

IQR (interquartile range), U-PBG (urine porphobilinogen)

An elevated U-ALA/creatinine level was recorded in 27 patients (20.9%), being moderate (5.1–20.0 mmol/mol) in 20 (15.5%) and high (≥ 20.1 mmol/mol) in 7 (5.4%) patients. Eighty-three patients (64.3%) had a U-ALA/creatinine level below the ULN (U-ALA normal), whereas data from 19 patients (14.7%) were missing (U-ALA unknown).

### Symptomatology

The different symptoms of the symptomatic AHP patients are shown in Table 3. The dominating symptom was abdominal pain, which was reported in 36 of 38 patients (94.7%) during an acute attack. Other common symptoms were nausea and vomiting (28.9%), and neurological disturbances (31.6%). Less common symptoms were constipation (15.8%), weakness (13.2%), diarrhea (10.5%), anxiety and depression (7.9%), and seizures (7.9%).

**Table 3:** Symptoms, precipitating factors, and co-morbidities for the study population

Symptoms	*n* (%)	Precipitating factor	*n* (%)	Co-morbidities	*n* (%)
Abdominal pain	36 (94.7%)	Unknown	15 (39.5%)	None	84 (65.1%)
Nausea, vomiting	11 (28.9%)	Alcohol	6 (15.8%)	Hypertension	19 (14.7%)
Weakness	5 (13.2%)	Stress	10 (26.3%)	Reduced eGFR	9 (7.0%)
Constipation	6 (15.8%)	Medication	7 (18.4%)	Headache	2 (1.6%)
Anxiety, depression	3 (7.9%)	Menstruation/hormonal	2 (5.3%)	Paresis	0 (0.0%)
Diarrhea	4 (10.5%)	Fasting	4 (10.5%)	Other	36 (27.9%)
Seizures	3 (7.9%)	Infection	10 (26.3%)		
Paresis, neurological disturbance	12 (31.6%)				

### Precipitating factors

Factors precipitating attacks in the symptomatic patients according to patients and physicians are shown in Table 3. Fifteen of the 38 symptomatic patients had unknown precipitating factors, while other patients had several potential precipitating factors of the attack. The most frequently reported precipitating factors were stress (10 patients), infection (10 patients) and medication (7 patients). Other precipitating factors reported were alcohol (6 patients), fasting (4 patients) and menstruation/hormonal (2 patients).

### Comorbidities

The comorbidities of the study population are shown in Table 3. Eighty-four (65.1%) patients had no known comorbidities, whereas the remaining 45 (34.9%) patients had at least one comorbidity. The reported comorbidities were hypertension (19 patients, 14.7%), reduced estimated glomerular filtration rate (eGFR) (9 patients, 7.0%), and headache (2 patients, 1.6%). Finally, 36 patients (27.9%) had other non-AHP-related comorbidities like diabetes, asthma, osteoporosis, rheumatoid arthritis, epilepsy, etc.

HCC is a known co-morbidity to AHP. Patients aged ≥ 50 years are offered a hepatic ultrasonography (ULS) either annually or biannually. In our population, 57 patients (44.2%) where in this group. Hereof, 49 patients (86.0%) had biannually ULS, and 7 patients (12.3%) had annually ULS check-up. One patient (1.7%) refrained having ULS. During the five-year study period, none of the 129 patients developed HCC.

### U-PBG subgroup analysis

Over the 5-year observation period, 38 out of 129 subjects experienced symptoms, resulting in an absolute risk of 29% (Table 2). When focusing on the 110 patients with known urinary excretion status, 36 had symptoms, resulting in an absolute risk of 33%, with absolute risks being 6% in normal excreters, 7% in moderate excreters and 19% in high excreters. However, we could demonstrate a relationship between baseline U-PBG/creatinine levels (i.e., outside attacks) and the relative risk of having symptoms. Thus, in high excreters the relative risk of having symptoms was 54% (21 of 39), as compared to 18% (7 of 40; *p* = 0.002) in normal excreters and 26% in moderate excreters (8 of 31; *p* = 0.034). Normal excreters and moderate excreters did not differ in terms of risk for symptoms. When comparing high vs. normal/moderate excreters, the risk for symptoms was 2.5-fold higher in high excreters. On the other hand, we observed no statistical differences between the three different U-PBG/creatinine excretion groups as regards number of hospitalizations (0.078) or number of treatments (0.16).

### U-PBG and U-ALA during acute attacks

**Fig. 2:**
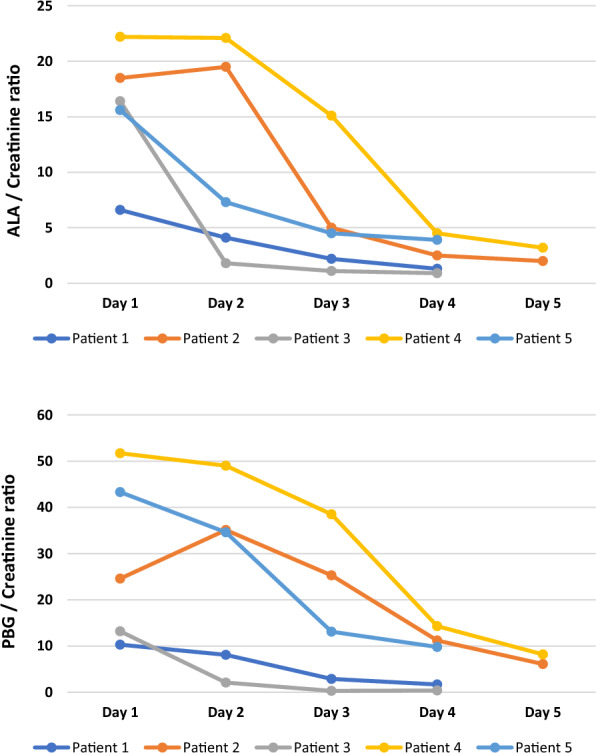
Progress, during an acute attack of AHP, of precursors urine porphobilinogen (U-PBG) and urine δ-aminolevulinic acid (U-ALA) after initiation of hemin treatment on Day 1

Administration of hemin rapidly reduces the urinary levels of porphyrin precursors in all treated patients, Fig. 2. However, at the time of the acute admission the relative increase in the urinary excretion of porphyria precursors varied considerably between patients. E.g., some patients had urinary precursor levels that were markedly elevated, whereas in others the increase was less dramatic. Figure 2 shows the variability between patients in the urinary excretion of ALA and PBG, respectively, during hemin treatment. Measurements are corrected for urinary creatinine concentrations.

## Discussion

This study provides information on the Danish population of patients with AHP. Such information is important to understand the disease manifestation of the AHP population and to recognize AHP patients in a clinical setting. Therefore, we searched our journals for information on patients with AHP during a 5-year period. This yielded 129 unique patients with AHP, with AIP being the dominant subtype (77.5%), and female sex the most prevalent sex (65.9%). Almost 30% of the AHP patients were symptomatic. According to hospitalizations and treatment in the five-year period, 15.5% of the patients had one or more hospitalizations and 10.9% had been treated with human hemin at least once. None of the 129 patients were diagnosed with HCC during the 5-years.

In general, AIP is the dominating AHP subtype, being reported to constitute between 57 and 96% of all AHP patients [4, 5, 15, 19]. Our cohort has a similar predominance of AIP (77.5%). Furthermore, we observed much lower frequencies of HCP (9.3%) and VP (13.2%), in alignment with previous findings, reporting HCP and VP in the range of 3–10% and 4–33%, respectively [4, 5, 15, 19]. The highest frequency of VP patients appears to be in France, Switzerland, and United Kingdom [4]. If we exclude these countries, the percentage of VP patients averages 16%; i.e., in the range observed in the present study. Thus, the Danish population of AHP patients resembles AHP populations in some other Western European countries. Finally, the distribution of patients with AIP, VP and HCP in Denmark appears to be stable as a similar distribution was reported in the first Danish AHP population study in 1980 [16].

The Danish prevalence of AHP (1.7:100,000) is lower than that found in Norway (7:100,000) [11] and Sweden (10:100,000) [20]. Likewise, the prevalence of symptomatic AIP found in our study (0.6:100,000) was substantially lower than that found in Norway (4:100,000) [11], but similar to the calculated prevalence (calculated on the basis of incidence from the participating countries) in Europe (0.5:100,000) [4]. The higher prevalence in Sweden and Norway is most likely caused by the founder effect originating from Northern Sweden, where the estimated prevalence is 1000:100,000 [20]. In addition, the awareness of AHP in Norway and Sweden may be greater due to the founder mutation, leading to a more extensive family screening and subsequently more cases being diagnosed [11]. Three other studies have shown a genetic frequency of mutations predisposing to AIP ranging from 1:1300 to 1:1785 [1], which indicate that the prevalence could be much higher than first thought. The prevalence of AHP in this investigation is based on clinical assessments and subsequent genetic investigations used to identify pathogenic variants in AHP-related genes. Although variants in the AIP-related *PEPT2* gene are associated with a higher risk of renal failure [21], genotypes and phenotypes are generally not correlated in AHP [22]. Moreover, the pathogenicity of some variants previously reported to be associated with AHP has been questioned [23], further limiting the prognostic impact of genetic variants in AHP.

Previous studies have reported that AHP is more commonly diagnosed in females [4, 5, 15, 19], a finding in line with our study, where 65.9% of the patients were female. In other studies, the percentage of female patients varies from 53–89%. Wang et al. [7] reported that females constituted approximately 90% of the symptomatic patients, whereas we found that 68.4% of the symptomatic patients were female. The reason why more female patients are experiencing symptoms is still not fully identified. Treatment with a gonadotropin-releasing hormone (GnRH)-agonist decreases the frequency of attacks in women suffering from menstrual-related acute porphyria attacks [24]. Moreover, the prevalence of symptoms generally decreases after the menopause in symptomatic female patients [24, 25]. This indicates that progesterone and estrogen play an important role in precipitating acute attacks, and thereby the female sex predominance.

In general, there is consistency between symptoms being reported by the Danish AHP patients experiencing acute attacks and those observed in other studies [5, 11, 19, 26]. In all studies, abdominal pain is the main symptom during attacks, being reported by 74–97% of all symptomatic patients. Although abdominal symptoms are a consistent finding, there is a difference in the percentage of the reported symptoms between the studies. This shows that even though patients are experiencing the same symptoms, the clinical picture can vary considerably between the patients.

In our study, 5 patients (13.2%) reported weakness, and 12 patients (31.6%) experienced other neurological symptoms. The symptoms and the progression of these symptoms vary among individual patients, but as described in earlier studies, the progression can be rapid with flaccid tetraplegia and respiratory paralysis appearing within days [7, 27]. Indeed, the neurological symptoms may simulate symptoms of Guillain-Barré [9] and there are case reports describing patients being falsely diagnosed with and treated for Guillain-Barré, but without improvement after several days of treatment [28, 29]. Finally, and of clinical relevance, 20–30% of the AHP population may present with signs of mental disturbances such as anxiety, depression, disorientation, hallucinations, and confusions [14].

The heme biosynthesis pathway can be induced by different precipitating factors, increasing the demand for heme and consequently, by feedback, accelerate the enzymatic activity [5]. Administration of drugs that induce the synthesis of cytochrome P-450 (particularly barbiturates and other related compounds), are known precipitating factors. Other known factors are female sex hormones, stress, alcohol, smoking, infection and fasting [14]. In our study, the use of drugs, stress and infections were reported as frequent precipitating factors. Drugs as a precipitating factor were reported more frequently in our study (18.4%) than in a South African study (10%) [26], but less common than in Norway (40%) [11] and USA (37%) [19]. The knowledge of precipitating factors is important, because the education of genetic carriers in avoidance of these factors is central to long-term management of AHP [7]. Equally important is to remember that some drugs can precipitate acute attacks. Therefore, we believe it is mandatory to check all prescribed medication when the patients is referred to the hospital (acutely or during routine checkup) and to check prescriptions of new drugs for any porphyrinogenic effects prior to prescription; e.g. via public databases such as https://www.drugs-porphyria.org/.

In our cohort, self-reported stress appeared as one of the most frequent precipitating factors. However, we believe it is difficult to clarify whether the self-reported stress was the cause of the acute attacks or if the acute attack caused stress. In this context, it is important to remember that acute attacks is known for causing neurological symptoms such as depression, confusion and anxiety [14]. Thus, stress may be a symptom rather than a precipitating factor.

Attacks of AHP may be provoked during weight-losing diets (i.e. calorie restriction) as well as following bariatric surgery [7]. Indeed, several cases [28–30] describe healthy obese patients, not known with a genetic mutation predisposing to AHP, experiencing severe abdominal pain and neurological symptoms after undergoing bariatric surgery such as gastric bypass or sleeve gastrectomy. Common to all cases are that patients were admitted to hospital after bariatric surgery with abdominal pain and weakness progressing to paresis. Subsequently, the patients demonstrated elevated porphyria precursors and received the diagnosis AHP. In one of the cases, gastric bypass was reversed, leading to relief of symptoms [28].

A known severe complication to AHP is HCC, and many of our patients have reported on family members having had HCC. In line with this, a study [15] found that the hazard ratio of developing HCC in AHP patients is 38.0 compared to a healthy reference population, making the authors advocating for regular surveillance in patients older than 50 years. In our cohort, 98.2% of all patients ≥ 50 years had biannual or annual screening for HCC. At the time of writing, none in our cohort have been diagnosed with HCC during the five-year period, which may be explained by the size of the study population, and particularly the short observation time. In contrast, Lissing et al. [15] identified 6.7% of their patients with HCC thereby stressing that continuous surveillance is important, as recently stated by Wang et al. in their clinical practice update [6].

Other chronic medical conditions include peripheral neuropathy, hypertension, and chronic kidney disease [19, 31]. The proportion of hypertension was lower in our study (14.7%), when compared to patients in US (43%) [19], whereas the proportion of chronic kidney disease (7.0%) was similar to what has been observed in Finland (5.7%) [31]. Here, the frequency in US has been reported to 29% [19].

Lissing et al. [15] studied urinary excretion of PBG outside of attacks (i.e., baseline) and they could demonstrate that 32% patients had a normal, 13% a moderate and 33% a high U-PBG/creatinine excretion. Compared to our cohort, the moderate U-PBG/creatinine excretion group differs. However, the upper normal limit (ULN) in that study [15] differs from the ULN in our study. The ULN and analytical methods can vary considerable among different laboratories [10], which makes it hard to find other studies with the exact same ULN as in our study. Nevertheless, it appears relevant to characterize the baseline excretion of PBG. Thus, in our cohort patients with a U-PBG/creatinine baseline level higher than 3.2 mmol/mol (i.e., outside of acute attacks) had a significantly higher risk of having acute attacks when compared to patients having a lower U-PBG/creatinine baseline level. This could be of potential clinical importance by focusing follow-up and education to patients most prone to develop acute attacks.

This retrospective description of the Odense AHP cohort houses limitations. AHP is a rare disease, which makes the estimate of its prevalence in Denmark uncertain. In addition, some Danish patients with AHP may not have been referred to our center, which could lead to an underestimation of the true prevalence. We obtained information on Danish AHP cases that were entered in medical records before and after the emergence of COVID-19. Although patient contact continued as phone calls and video contacts or as physical attendance when deemed necessary, we cannot exclude the possibility that the pandemic may have influenced occurrence and characteristics of self-reported symptoms, prevalence of attacks and admissions as well as the frequency of biochemical tests. At last, due to legal limitations, we were only able to perform a 5-year retrospective search in the hospital’s journal system. Thus, we may have missed admissions at other hospitals. However, our study also has some strengths. The Department of Endocrinology at Odense University Hospital serves as the national center for patients with AHP in Denmark. Accordingly, we believe our outpatient clinic is housing the vast majority of Danish AHP patients and that data therefore are representative for Denmark.

In conclusion, this study found the cohort of Danish AHP patients to be like previously described patient populations in international studies. The most common type of AHP was AIP, and females dominated both overall and in the symptomatic patient group. Furthermore, only about half of the patients experiencing symptoms needed hospital admission and even fewer needed treatment with human hemin to get their acute attack under control. At last, the urinary PBG/creatinine concentration appears to be of prognostic value, as is predicts the risk of having symptoms, but not to the risk of hospitalization or need for treatment.

## Data Availability

We have extracted data from patient journals and subsequently stored all at secure- and access-logged serves at the hospital. Because data contains journal information, they are not accessible for third party.

## Abbreviations

ALAS: ALA synthase
ALA: Aminolevulinic acid
COPRO: Coproporphyrin
COPROgen III: Coproporphyrinogen III
CPOX: Coproporphyrinogen oxidase
ADP: Delta-aminolevulinic acid (ALA) dehydratase deficiency porphyria
eGFR: Estimated glomerular filtration rate
GnRH: Gonadotropin-releasing hormone
HCC: Hepatocellular carcinoma
HCP: Hereditary coproporphyria
HMB: Hydroxymethylbilane
HMBS: Hydroxymethylbilane synthase
PBG: Porphobilinogen
PROTO: Protoporphyrin
PROTOgen IX: Protoporphyrinogen IX
PPOX: Protoporphyrinogen oxidase
ULS: Ultrasonography
ULN: Upper normal limit
URO: Uroporphyrin
VP: Variegate porphyria
